# Radiolabeling Strategies of Nanobodies for Imaging Applications

**DOI:** 10.3390/diagnostics11091530

**Published:** 2021-08-25

**Authors:** Jim Küppers, Stefan Kürpig, Ralph A. Bundschuh, Markus Essler, Susanne Lütje

**Affiliations:** Department of Nuclear Medicine, University Hospital Bonn, 53127 Bonn, Germany; stefan.kuerpig@ukbonn.de (S.K.); ralph.bundschuh@ukbonn.de (R.A.B.); markus.essler@ukbonn.de (M.E.); susanne.luetje@ukbonn.de (S.L.)

**Keywords:** nanobodies, labeling strategies, positron emission tomography, single photon emission computed tomography, radiometals, radiohalogens, molecular imaging

## Abstract

Nanobodies are small recombinant antigen-binding fragments derived from camelid heavy-chain only antibodies. Due to their compact structure, pharmacokinetics of nanobodies are favorable compared to full-size antibodies, allowing rapid accumulation to their targets after intravenous administration, while unbound molecules are quickly cleared from the circulation. In consequence, high signal-to-background ratios can be achieved, rendering radiolabeled nanobodies high-potential candidates for imaging applications in oncology, immunology and specific diseases, for instance in the cardiovascular system. In this review, a comprehensive overview of central aspects of nanobody functionalization and radiolabeling strategies is provided.

## 1. Introduction

**Figure 1:**
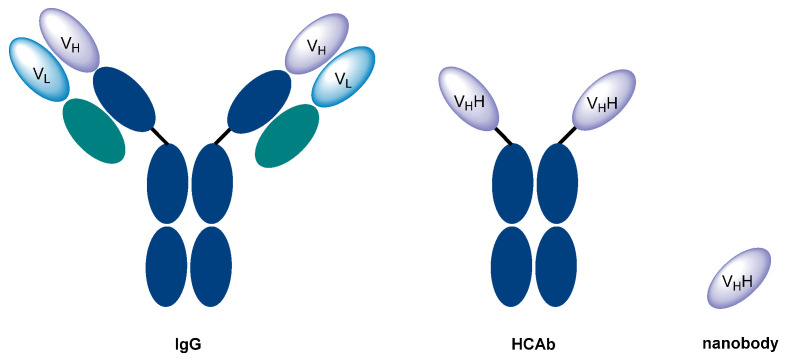
Schematic representation of conventional immunoglobulin G’s (IgGs), heavy chain-only antibodies (HCAbs) and nanobodies. V_H_, variable heavy; V_L_, variable light; V_H_H, V_H_ of HCAbs.

Nanobodies (V_H_Hs) represent recombinant single-domain variable fragments of heavy-chain-only antibodies (HCAbs), which themselves are obtained from species of the *Camelidae* family (Figure 1) [1]. With a molecular weight of 12–15 kDa, nanobodies are considered the smallest naturally occurring antigen-binding fragments [2]. They exhibit many beneficial features such as good water-solubility and (thermo)stability, high affinity and specificity as well as low immunogenicity, predestining them as excellent probes for molecular imaging applications [1,3,4]. A major advantage compared to conventional immunoglobulin G (IgG) antibodies is their rapid pharmacokinetics [5]. Due to their small size, nanobodies can reach their binding sites on the target tissues efficiently and quickly after injection, while the unbound fraction is rapidly cleared from the blood stream through renal elimination, potentially leading to high target-to-background ratios [6,7].

Clinically, molecular imaging allows for noninvasive diagnosis of various ailments, disease-monitoring and therapy follow-up, as well as for patient selection and stratification at an early stage [7,8]. In order to depict (patho)physiological processes in vivo, a molecular tracer consisting of a targeting moiety and a detection label is appropriately administered into the patient’s body [6]. While the former accomplishes the direction and specific accumulation of the probe, the latter enables the visualization of the tissue of interest [7]. For nuclear imaging purposes, a gamma-emitting isotope is used for single photon emission computed tomography (SPECT), while positron-emitting isotopes are used for positron emission tomography (PET). Typical SPECT isotopes comprise technetium-99 m and indium-111, while common PET isotopes include zirconium-89, copper-64, gallium-68 and fluorine-18. Since both techniques have their advantages in imaging, it is important to evaluate labeling for gamma emitters as well as positron emitters. Quantification in PET is easier than in SPECT imaging, where more individual calibration techniques are necessary [9]. SPECT, however, allows multi tracer imaging, as different energy windows can be scanned at the same time [10]. Clinical PET scanners also show a higher spatial resolution compared to clinical SPECT in most cases [11]; this is different in small animal imaging as spatial resolution of SPECT is not limited by the positron range, which is an intrinsic physical limit for the spatial resolution of PET.

It is important that the pharmacokinetic properties of the targeting vehicle are perfectly coordinated with the half-life of the radioisotope. Thus, due to their relatively slow pharmacokinetics, full-size IgG antibodies need to be combined with longer-lived radionuclides, such as zirconium-89 or indium-111 [12]. Upon administration, these radiolabeled antibodies require longer waiting periods for imaging, along with extended exposure to ionizing radiation for the patients. Such a longer accumulation time might affect the stability of the radiotracer and result in disintegration, leading to changes in the biodistribution profile and, via a misinterpretation of the scan, to a false diagnosis. Accordingly, they are less favorable for clinical applications. Conversely, nanobodies with more rapid pharmacokinetics attached to short-lived radionuclides, such as gallium-68 or fluorine-18, represent ideal imaging radiotracers [3].

In order to attain such tracers, the radiolabel may either be introduced into the single peptide chain of the nanobody as part of a prosthetic group, relating to radiohalogens (e.g., fluorine-18), or by means of complexation, concerning primarily radiometals (e.g., gallium-68) [12]. Direct radiohalogenation can require harsh reaction conditions, such as high temperatures and non-aqueous conditions, which are incompatible with nanobodies. Therefore, indirect labeling by using radiohalogen-containing prosthetic groups is most commonly performed for such antigen-binding proteins, although the procedure for synthesizing and purifying these radiolabeled groups requires extra time [3,12]. Contrary to this, radiometal labeling via chelation is usually conducted at the very end, immediately prior to application. Accordingly, the nanobody needs to be pre-modified with a chelating moiety in order to coordinate the radio-cation. Such a chelator can either be a synthesized organic molecule or of proteinogenic nature. Another way to introduce radiometals to nanobodies is via a heteroleptic complex, which is usually formed prior to attachment.

After intravenous administration, these nanobody-based radiotracers are rapidly cleared from the blood circulation through glomerular filtration due to their small size, followed by reabsorption in the proximal tubule, leading to a longer retention time in the renal cortex, which presents diagnostic as well as health concerns [7]. On the one hand, the associated intense renal signals impede imaging of molecular targets in close proximity to the kidneys. On the other hand, a long-term exposure with these tracers and their radio-catabolites implies a certain undesired nephrotoxicity. This refers especially to radio-catabolites derived from radiometal-labeled nanobodies, since radiohalogenated (fluorinated or iodinated) catabolites are usually hydrophobic and therefore rapidly excreted via the urine. In order to diminish the renal reabsorption of the tracers, a few techniques have been implemented. Since megalin/cubilin receptors in the proximal tubule play a decisive role for the reuptake, co-administration of the plasma expander gelofusin or positively charged amino acids, such as arginine or lysine, can prevent the recovery through competition [7,13]. Apart from this, the tracer can be chemically modified, either by a cleavable linker (e.g., a renal brush border enzyme (BBE)-cleavable linker) inserted between the targeting moiety and the label so that an easier excretion of the radioactive metabolites into the urine is accomplished [14], or by an increase in the negative charge of the labeled entity, evoking a stronger electrostatic repulsion with the negatively charged proximal tubular cell surface [15].

**Figure 2:**
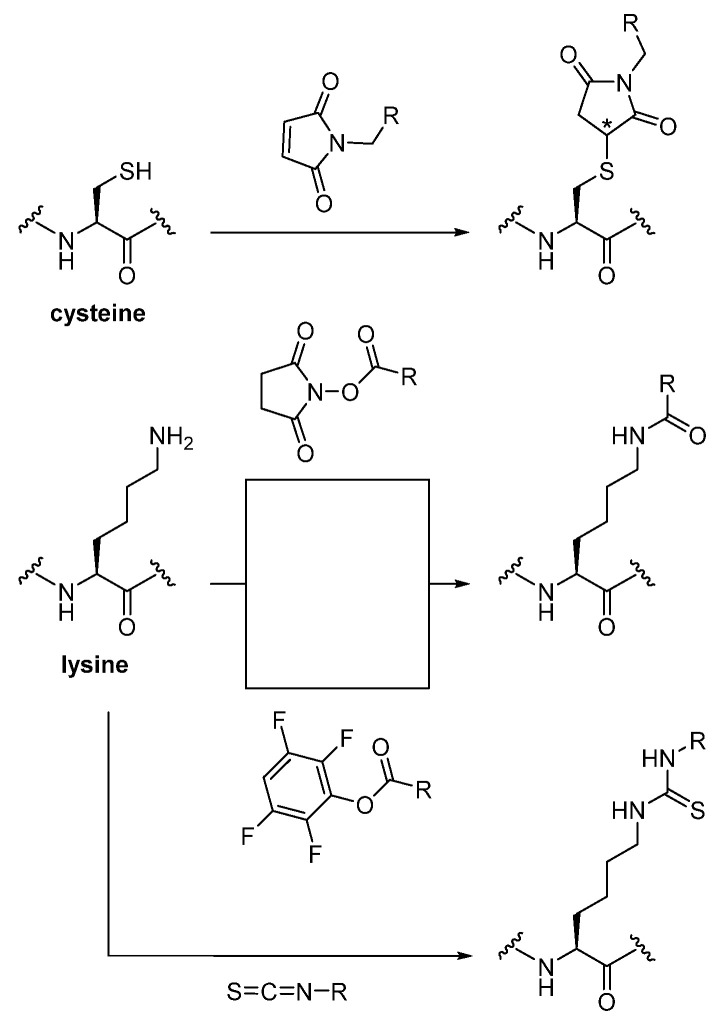
Common bioconjugation reactions for the attachment of radiolabels on cysteine and lysine residues within the nanobody [12]. Asterisk (*) indicates a chiral center with an undefined ratio of the two stereoisomers.

Among the canonical amino acids within the nanobody, cysteine and lysine are most commonly addressed for radiolabeling [12]. While the thiol function of the former rapidly forms a thioether with a maleimide moiety, the primary ε-amino residue of the latter is easily acylated via activated esters or converted into stable thioureas through isothiocyanates, all of which enable the attachment of chelators or prosthetic groups to the nanobody (Figure 2). Whether their installation to its amino acid sequence is carried out randomly or site-specifically determines whether the radiotracer is obtained as a heterogenous or a homogenous product [7]. Unselective (random) conjugation as a classical strategy is convenient and has proven to be valuable; however, it can easily lead to hindered target recognition when the label is inserted within or in close proximity to the antigen binding site [16,17,18]. In order to better control the tracer conception, selective labeling at a specific attachment site is the favored approach. This review is aimed at providing an overview of the different synthetic strategies for radiolabeling nanobodies, which have been employed during the past decade and up to today.

## 2. Radiolabeling Strategies of Nanobodies

### 2.1. Radiohalogens

Radioiodines are well established in nuclear medicine [19], and among the clinically used isotopes, iodine-125 and iodine-131 can be applied for both diagnostic and therapeutic purposes [20]. Their gamma emission enables SPECT imaging, while additional radiation allows for disease treatment [19]. However, they both possess a relatively long half-life (59.6 days for iodine-125 [21]; 8.02 days for iodine-131 [22]), which is less desirable for imaging applications of nanobody-based radiotracers. A much better radiohalogen in this regard is the widely used PET imager fluorine-18 (t_1/2_ = 110 min) [23], which is highly valued for its high positron yield on the one hand, leading to higher sensitivity, and its low positron energy on the other hand, optimizing resolution in imaging [12,24].

#### 2.1.1. Direct Radiohalogenation

**Figure 3:**
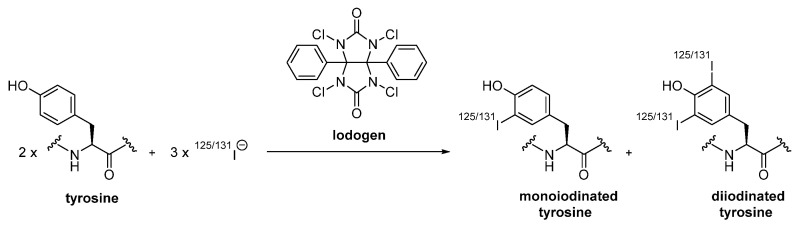
Installation of iodine-125 or iodine-131 into tyrosine residues of proteins and peptides via Iodogen [31].

While direct radiofluorination of nanobodies implies incompatible harsh reaction conditions, direct radioiodination using a well-established method with Iodogen (1,3,4,6-tetrachloro-3α,6α-diphenyl-glycoluril), a water-insoluble oxidant that is applied in order to minimize any protein damage through oxidation [25], has been performed by Pruszynski et al. [26,27]. Therein, the 5F7 nanobody, which specifically binds to the same epitope on the human epidermal growth factor receptor type 2 (HER2) as the known antibodies trastuzumab and pertuzumab, has been labeled with either iodine-125 or iodine-131, on constituent tyrosine residues of the nanobody’s peptide chain, by electrophilic substitution. The phenolic hydroxyl group of tyrosine with its electron donating ability directs the positively charged iodine species obtained by iodide oxidation with Iodogen in the ortho position of the aromatic ring [28,29], yielding a heterogenous mixture, wherein several tyrosines of the nanobody are either mono- or disubstituted (Figure 3). For internalizing targets, such as HER2, direct radioiodination methods are less appropriate, due to compromised cumulative radioactivity within the cell as a result of rapid excretion of the primary radiolabeled catabolites, e.g., iodotyrosines and free iodide, obtained by lysosomal degradation [26,30].

#### 2.1.2. Indirect Radiohalogenation

##### Prosthetic Groups

**Figure 4:**
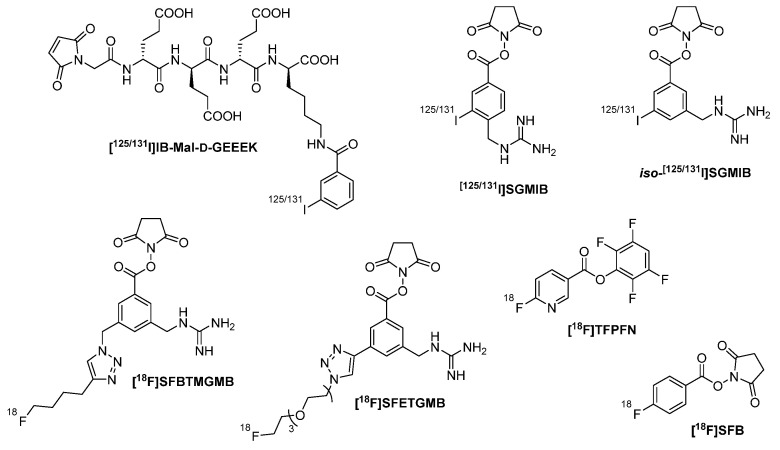
Radiohalogenated prosthetic groups which have been applied to nanobodies in a single final conjugation reaction.

The same anti-HER2 nanobody (5F7) has been labeled with iodine-125 or iodine-131 by introducing *N*^ε^−(3-[^125/131^I]iodobenzoyl)-Lys^5^-*N*^α^-maleimido-Gly^1^-GEEEK ([^125/131^I]-IB-Mal-d-GEEEK) (Figure 4) to sulfhydryl groups, which have been priorly installed on primary amines of the nanobody’s amino acid sequence [26,27]. These include the ε-amino functionality of several lysines, but also the *N*-terminal α-amino residue of the nanobody’s peptide chain. Upon addition of the cyclic electrophile 2-iminothiolane, the nucleophilic amino groups are converted into amidines with a free thiol moiety as a result of ring opening [32]. In a Michael-type addition reaction, the thiol-derivatized nanobody can be further conjugated with the maleimide function of [^125/131^I]-IB-Mal-d-GEEEK. The structure of this prosthetic group is based on the peptide sequence Gly-d-Glu-d-Glu-d-Glu-d-Lys-OH, in which the *N*-terminal amino group of glycine is part of the maleimide and the ε-amino group of lysine is acylated by 3-[^125/131^I]iodobenzoic acid. The three glutamic acids as well as the *C*-terminal lysine, all of which bear free carboxylic acid moieties, provide high polarity to the molecule. Apart from achiral glycine, all the amino acids within the pentapeptide are d-configured, rendering the sequence unsusceptible for proteolysis. Accordingly, such a prosthetic group is especially suited for radiolabeling structures that undergo intracellular processing, since it resists lysosomal digestion leading to a reduced efflux and thus to an increased retention inside the cell. Another way to trap radioactivity intracellularly is to apply radiohalogenated aromatic acylation agents comprising substituents, which remain charged at lysosomal pH. These also include *N*-succinimidyl 4-guanidinomethyl-3-[^125/131^I]iodobenzoate ([^125/131^I]SGMIB) as well as its isomer *N*-succinimidyl 3-guanidinomethyl-5-[^125/131^I]iodobenzoate (*iso*-[^125/131^I]SGMIB), differing from each other only in the substitution position of the highly basic guanidinomethyl moiety (Figure 4) [27,33]. Both prosthetic groups with their succinimide ester functionalities have been conjugated randomly to primary amines within 5F7. The conjugation reaction is usually performed in the range of pH 8–9, at which the *N*-terminal α-ammonium (pKa~8) is largely deprotonated, while the ε-ammonium (pKa~10) is mostly protonated, rendering it less reactive towards the carbonyl carbon of the activated ester. For this reason, it is likely to obtain a mixture of radiotracers, in which only one or a few among all the existing amino groups within the nanobody are radiolabeled. Indirect radioiodination with [^125^I]SGMIB has been further conducted in order to label another anti-HER2 nanobody, 2Rs15d, binding to a target site distinct from that of 5F7 and thus allowing for imaging patients that are subjected to trastuzumab or pertuzumab therapy [34].

**Figure 5:**
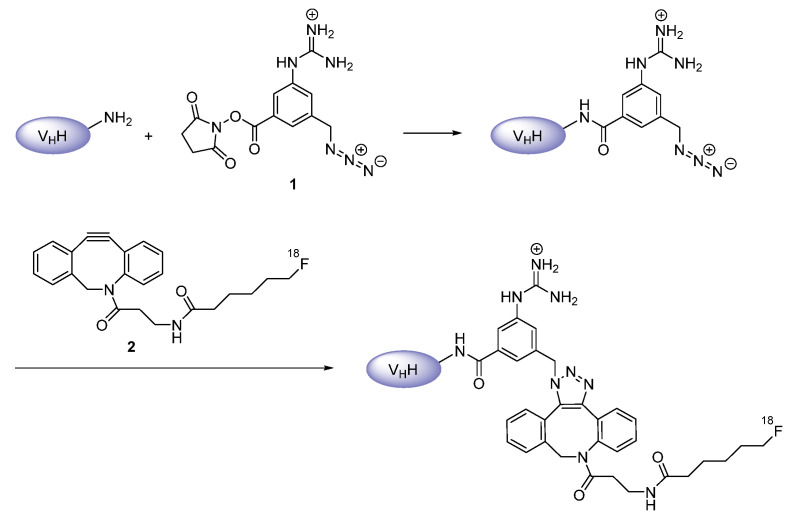
Pre-derivatization on primary amines of the nanobody with **1**, followed by ^18^F-introduction via **2** in a strain-promoted azide-alkyne cycloaddition reaction [36].

Furthermore, both nanobodies were also envisaged for indirect radiofluorination. In order to obtain ^18^F-containing prosthetic groups closely related to the guanidinomethyl-incorporating SGMIB, *N*-succinimidyl 3-((4-(4-[^18^F]fluorobutyl)-1*H*-1,2,3-triazol-1-yl)methyl)-5-(guanidinomethyl)benzoate ([^18^F]SFBTMGMB) and its analog *N*-succinimidyl 3-(1-(2-(2-(2-(2-[^18^F]fluoroethoxy)ethoxy)ethoxy)ethyl)-1*H*-1,2,3-triazol-4-yl)-5-(guanidinomethyl)benzoate ([^18^F]SFETGMB) (Figure 4) were synthesized through a multiple-step procedure involving copper(I)-catalyzed azide-alkyne cycloaddition (CuAAC) for the ^18^F-introduction almost at the very end, shortly prior to conjugation to primary amines of the nanobodies’ peptide chain [30,34,35]. Nevertheless, the radiofluorinated nanobodies were achieved in very low overall decay-corrected radiochemical yields. Besides, Zhou et al. applied a different synthetic protocol, in which the click reaction was used for the attachment of the radiolabel to the nanobody [36]. In the first step, primary amines within 2Rs15d were pre-modified through an acylation reaction using *N*-succinimidyl 3-(azidomethyl)-5-(guanidinomethyl)benzoate (**1**) (Figure 5). Subsequently, the ^18^F-labeled aza-dibenzocyclooctyne derivative (**2**) was employed in order to conduct a strain-promoted azide-alkyne cycloaddition (SPAAC), yielding the desired radiotracer. Such copper-free click chemistry is especially suited for proteins, due to the avoidance of potential complex formation. Despite reducing the total radiosynthesis time by this approach, the overall radiochemical yield was still not satisfactory, tracing back to the lower reaction yield of SPAAC compared to CuAAC. Accordingly, improvements for both strategies need to be developed before application on a routine basis.

**Figure 6:**
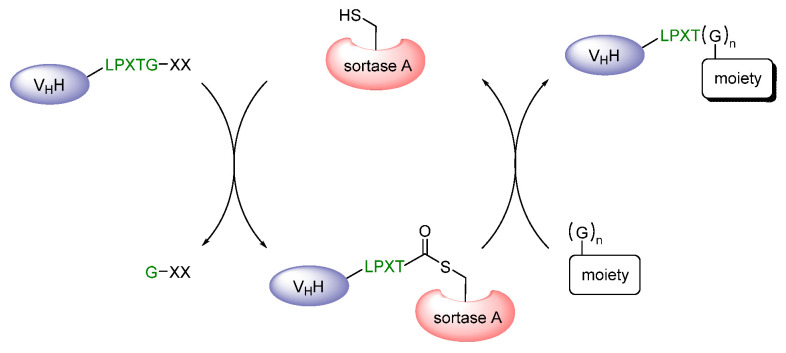
Reaction mechanism of the sortase A-mediated transpeptidation for labeling nanobodies site-specifically at the *C*-terminus [44]. LPXTG, sortase A-recognition motif; X, any amino acid except cysteine; G, glycine; SH, thiol function of the active site cysteine; C=O, carbonyl carbon of threonine.

A certain limitation of these indirect methods for radiofluorinating the anti-HER2 nanobodies was the high kidney uptake of the radiotracers. Therefore, another ^18^F-labeled prosthetic agent, namely 2,3,5,6-tetrafluorophenyl 6-[^18^F]-fluoronicotinate ([^18^F]TFPFN) (Figure 4), has been applied in order to radiolabel 2Rs15d and 5F7, respectively [37]. Its structure is based on a pyridine ring bearing the radiofluorine in position 6 and the carboxylic acid functionality in position 3, which in turn is activated as a 2,3,5,6-tetrafluorophenyl ester, enabling the random conjugation to primary amines within the nanobodies’ amino acid sequence. This molecule is closely related to the most commonly used prosthetic group for ^18^F-labeling, *N*-succinimidyl-4-[^18^F]-fluorobenzoate ([^18^F]SFB) (Figure 4), differing from it only by the aromatic system, which herein is a benzene ring, and by the activated ester, i.e., a succinimide ester. Consequently, the attachment to both anti-HER2 nanobodies was conducted analogously [3,30]. Indeed, the introduction of these two very similar prosthetic groups to 5F7 and 2Rs15d has proven valuable in terms of a lower renal uptake of the resulting radiotracers. Apart from HER2, [^18^F]SFB has been additionally used to radiofluorinate the two mouse-human cross-reactive nanobodies MMR 3.49 and cAbVCAM-1−5, specifically targeting the macrophage mannose receptor (MMR) and the vascular cell adhesion molecule (VCAM)-1, respectively [38,39]. Rashidian et al. described a very elegant method for radiofluorinating nanobodies (VHHDC13, VHH7, VHHDC8), which were specifically directed against the mouse cell surface marker CD11b or the mouse class II major histocompatibility complex (MHC) [40,41,42]. The two anti-class II nanobodies, VHH7 and VHHDC8, recognize a closely related epitope, but differ from each other in their affinity towards the target, which is 3–4 fold higher for VHHDC8 than for VHH7 [40]. These two as well as the anti-CD11b nanobody VHHDC13 were engineered in a way that they bore a sortase A-recognition motif (sortag), enabling *C*-terminal site-specific conjugation. The sortag itself embodies the oligopeptide Leu-Pro-Xxx-Thr-Gly (LPXTG), in which Xxx is any amino acid besides cysteine, and glycine is not the final *C*-terminal amino acid of the whole protein chain [43,44,45]. Sortase A is an enzyme found in *Staphylococcus aureus* that catalyzes transpeptidation reactions. Upon recognition, the thiol (-SH) of the transpeptidase’s active-site cysteine nucleophilically attacks the carbonyl carbon (C=O) of the sortag’s threonine, forming an acyl-enzyme intermediate (Figure 6). Subsequently, the carbonyl carbon of the thioester is nucleophilically attacked by the amino group of a different oligoglycine which is present in molar excess, preventing the reverse reaction.

**Figure 7:**
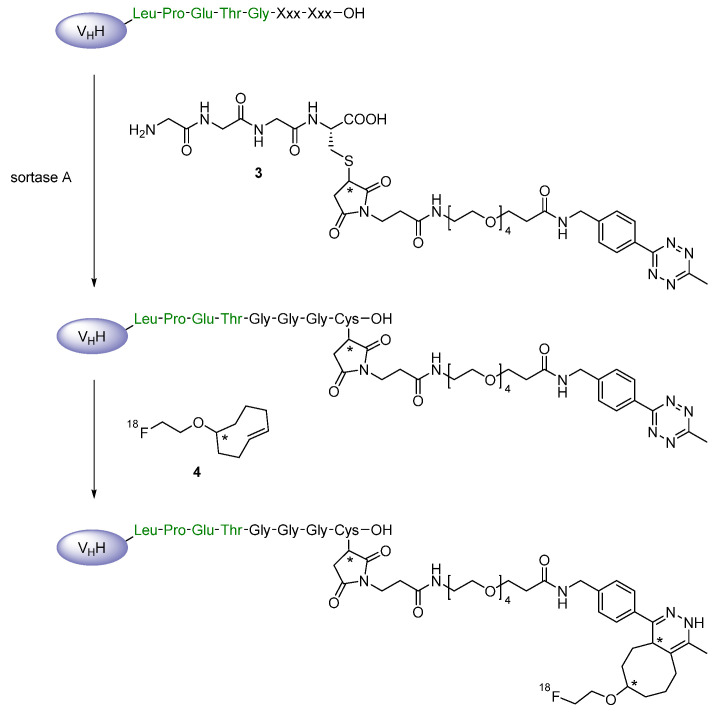
Site-specific insertion of **3** at the nanobody’s *C*-terminus via sortase A and subsequent inverse-electron demand Diels-Alder (IEDDA) cycloaddition with **4**.

**Figure 8:**
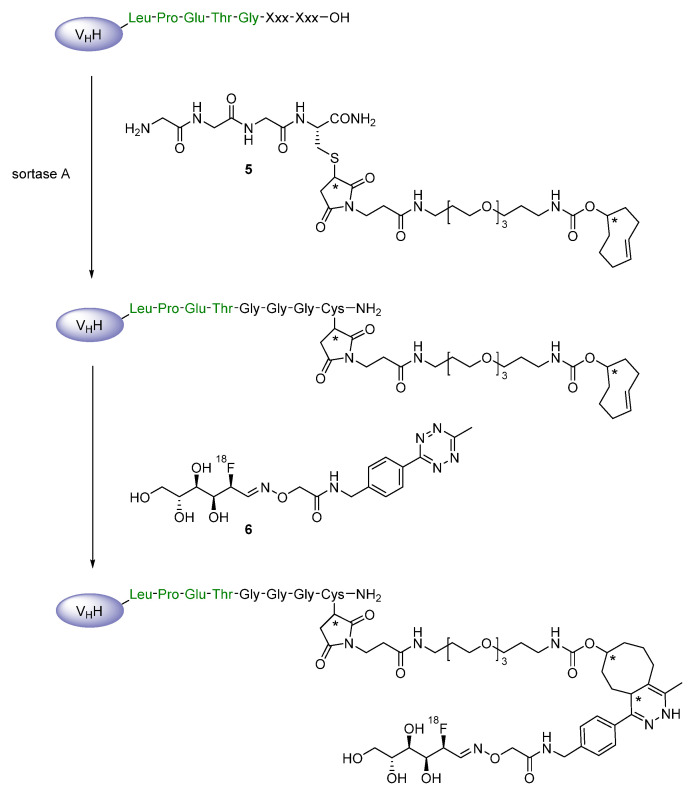
Sortase A-mediated *C*-terminal introduction of **5**, followed by ^18^F-labeling with **6** in an IEDDA reaction.

**Figure 9:**
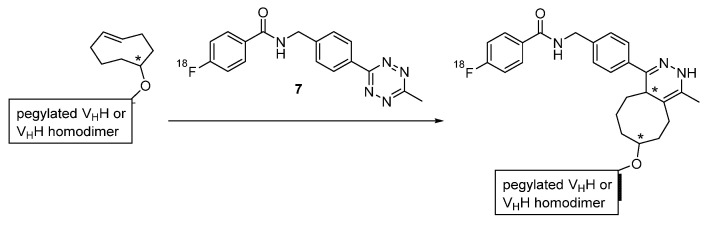
^18^F-labeling of polyethylene glycol-functionalized or homodimeric nanobodies with **7** through IEDDA cycloaddition [42].

Such *N*-terminal oligoglycines decorated with specific functionalities have been used by Rashidian et al. in order to site-specifically modify the three nanobodies so that an inverse-electron demand Diels-Alder (IEDDA) cycloaddition between a tetrazine and a *trans*-cyclooctene moiety could be employed for the installation of ^18^F-containing prosthetic groups [40,41,42]. Firstly, the linker-connected triglycine-methyltetrazine compound **3** has been applied to introduce the tetrazine substructure at the *C*-termini of VHH7 and VHHDC13 via sortase reaction, followed by the addition of the radiofluorinated *trans*-cyclooctene derivative **4** to obtain the desired radiotracers through IEDDA reaction (Figure 7) [41]. In a similar procedure, the *trans*-cyclooctene function has been site-specifically inserted into VHH7 and VHHDC8 through their sortags by using the respective triglycine **5**, while the tetrazine moiety for the click reaction was part of the ^18^F-incorporating prosthetic agent **6** (Figure 8), which itself was obtained via an oxime ligation reaction with commercially and widely available 2-deoxy-2-[^18^F]fluoro-d-glucose [40]. This approach has undergone further development to enable the indirect radiofluorination of homodimeric and pegylated forms of VHHDC13 and VHHDC8 with the radiolabeled tetrazine **7** (Figure 9) [42]. Apart from the adjustment, the initial synthesis strategy using compounds **3** and **4** has been employed to three further recombinant nanobodies (A12, B3 and H11), from which A12 and B3 target the mouse programmed death ligand 1 (PD-L1) and H11 addresses the mouse cytotoxic T lymphocyte antigen (CTLA)-4 [46,47].

##### Chelation

**Figure 10:**
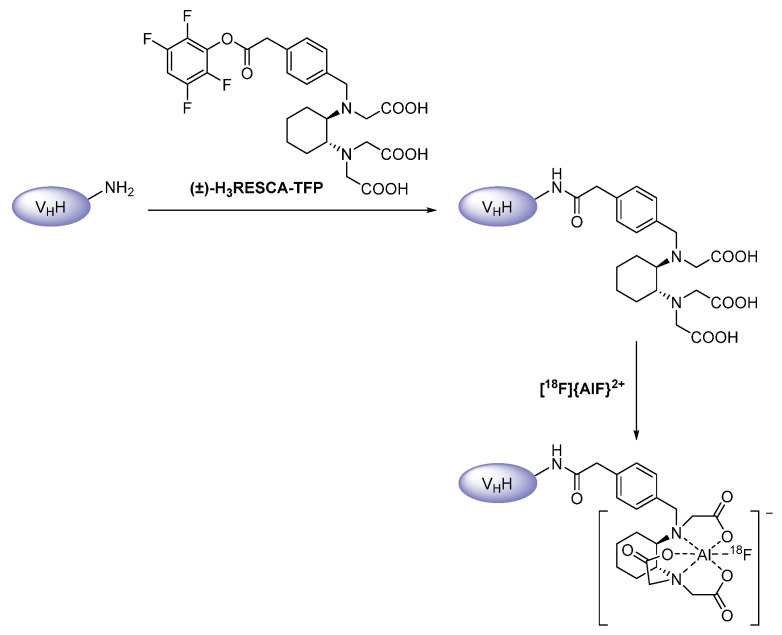
Conjugation of (±)-H_3_RESCA-TFP to primary amines of the nanobody, followed by Al^18^F-labeling [49,50].

**Figure 11:**
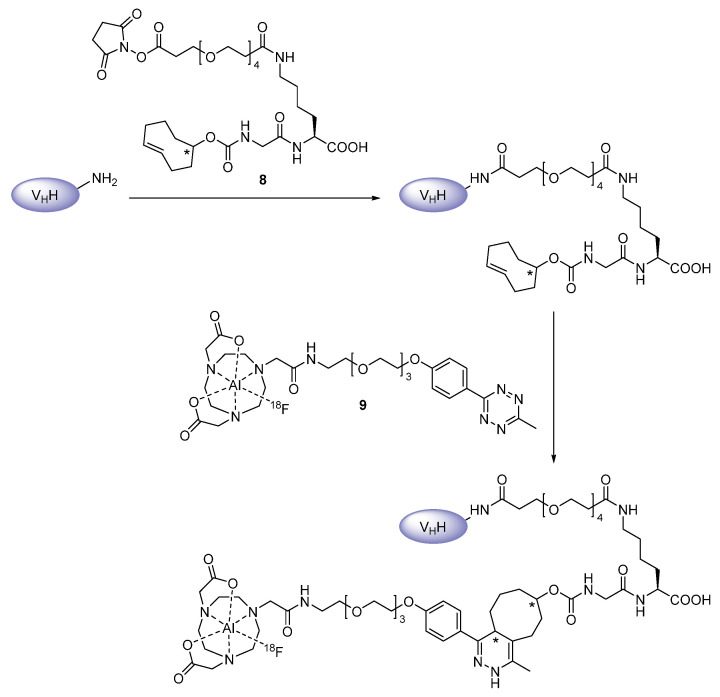
Pre-derivatization on primary amines of the nanobody with **8**, followed by ^18^F-labeling with **9** via IEDDA reaction [15].

Bifunctional chelating agents are very common for radiometal labeling, but their application can also be extended to radiofluorines. For this purpose, the radiofluoride is attached to a suitable metal, in particular aluminum, which itself is bound to an appropriate chelator conjugated to a targeting vehicle, altogether resulting in a stable complex [48]. Such an Al^18^F-labeling strategy has also been applied to the three nanobodies 2Rs15d, cAbVCAM-1−5 and NbV4m119, with the latter addressing the complement receptor of the immunoglobulin superfamily (CRIg) expressed on Kupffer cells [15,49,50,51]. Cleeren et al. established a new restrained complexing agent (RESCA) in order to facilitate the chelation reaction with aluminum mono[^18^F]fluoride ([^18^F]{AlF}^2+^) at room temperature, which is particularly suited for heat-sensitive biomolecules, e.g., nanobodies (Figure 10) [49,50]. Prior to complexation, the acyclic pentadentate chelator (±)-H_3_RESCA was randomly introduced to primary amines of the two nanobodies via the activated form, bearing a 2,3,5,6-tetrafluorophenyl ester ((±)-H_3_RESCA-TFP). Zhou et al. followed a more lengthy but elegant approach to radiolabel the 2Rs15d nanobody with aluminum mono[^18^F]fluoride via the macrocycle 1,4,7-triazacyclononane-*N*,*N*′,*N*″-triacetic acid (NOTA) [15]. By using the IEDDA reaction in tandem with a renal BBE-cleavable glycine-lysine (GK) linker in the prosthetic moiety, a good labeling yield as well as a low uptake of ^18^F-activity in the kidneys was achieved. For this purpose, the *trans*-cyclooctene moiety was introduced to 2Rs15d by randomly reacting the nanobody’s primary amines with the succinimide ester of TCO-GK-PEG_4_-NHS (**8**), which was then clicked to [^18^F]AlF-NOTA-PEG_3_-methyltetrazine (**9**) to yield the final tracer (Figure 11). Short polyethylene glycol (PEG) chains consisting of three and four units, respectively, have been implemented not only to further reduce the kidney uptake, but also to provide structural flexibility to the molecule in order to enable an enhanced enzyme accessibility to the cleavable GK linker. Although low kidney activity levels were accomplished, tumor uptake was impaired, which was not related to the tracer’s [^18^F]AlF-NOTA moiety, since its replacement by a prosthetic group did not remedy the problem [52]. All in all, compared to the other indirect radiofluorination strategies, the Al^18^F-chelation technique allows for higher radiochemical yields in a substantial shorter synthesis time, which is a key advantage of chelator-based radiolabeling methods [15,49,50].

### 2.2. Radiometals

Radiometal labeling is also based on chelation and represents an attractive alternative to radiohalogenation [53]. Owing to its simplicity, reproducibility and high efficiency, this method can be easily implemented in clinical routine. Among the common PET radiometals, gallium-68 is very convenient because of its simple, cyclotron-independent production via germanium-68/gallium-68 generators [12,54]. It also exhibits a short half-life of 67.7 min, along with a low positron energy as well as a high positron yield; the latter reflects a major decay through positron emission. In contrast, copper-64 and zirconium-89 constitute radiometals with supplemental alternative decay pathways, requiring higher administration doses because of lower sensitivity. Additionally, both radionuclides have much longer half-lives (12.7 h for copper-64 [55]; 78.4 h for zirconium-89 [56]), rendering them less appropriate for nanobody radio imaging. This also applies to the gamma-emitting radiometals indium-111 and lutetium-177, which possess a half-life of 67.2 h [57] and 6.65 days [58], respectively. Furthermore, lutetium-177 is mainly applied for therapeutic purposes due to the emission of low-energy β-minus particles [59]. Hence, much more suitable for SPECT in this context is the shorter lived technetium-99m (*t*_1/2_ = 6.02 h [60]), which can also be easily obtained from widespread molybdenum-99/technetium-99 m generators [61].

#### 2.2.1. Synthetic Chelators

The majority of chelating agents are produced by means of organic chemistry. The applied bifunctional chelators (BFCs) bear a chemically reactive functional group for attachment to the targeting vehicle on the one hand, and a metal binding moiety for sequestration of the metallic radionuclide on the other hand [13].

##### Macrocyclic

**Figure 12:**
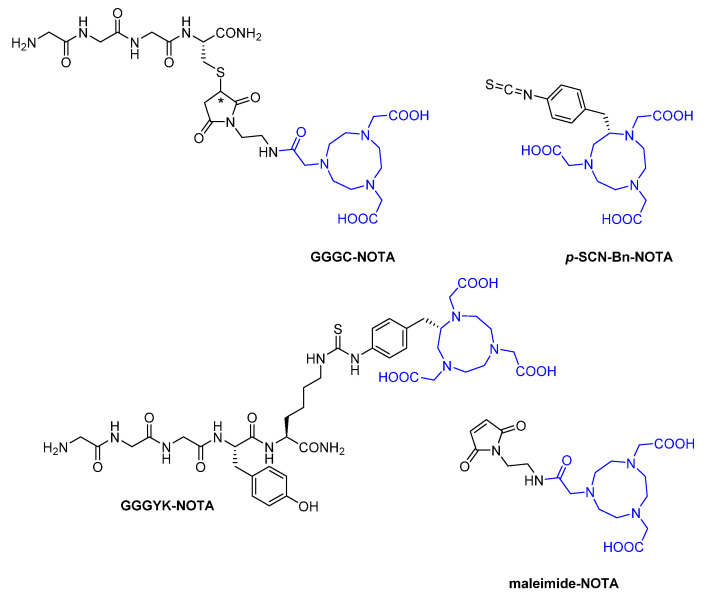
1,4,7-Triazacyclononane-*N*,*N*′,*N*″-triacetic acid (NOTA)-based synthetic chelators applied to nanobodies for radiometal labeling. NOTA substructure is highlighted in blue.

Among the different available macrocyclic chelators, almost exclusively NOTA has been used for radiolabeling nanobodies. This hexadentate ligand is especially suited for such heat-labile proteins, due to the rapid and highly efficient complexation of copper-64 and gallium-68 at room temperature [54]. Site-specific labeling with copper-64 has been conducted by introducing GGGC-NOTA (Figure 12) to the *C*-termini of several nanobodies (B3, VHH7, VHHDC13, VHH4, NJB2) via sortase A-mediated reaction, followed by radiometal chelation, as common in post-labeling strategies [41,46,62,63]. Its molecular structure is based on the tetrapeptide sequence H-Gly-Gly-Gly-Cys-NH_2_, in which the cysteine’s thiol function is covalently linked to maleimide-NOTA. Unlike the anti-mouse class II MHC nanobody VHH7, VHH4 targets human class II MHC products [62]. NJB2 is a mouse-human cross-reactive nanobody specific to an alternatively spliced domain of fibronectin expressed in disease extracellular matrix and neo-vasculature [63]. For random ^64^Cu-labeling, different nanobodies (Lox1.14, MMR 3.49, cAbVCAM-1−5) have been decorated with 2-*S*-(4-isothiocyanatobenzyl)-NOTA (*p*-SCN-Bn-NOTA) (Figure 12) [64], a BFC that bears an isothiocyanate functionality, enabling the reaction with primary amines of the amino acid sequence. Apart from Lox1.14 targeting the lectin-like oxidized low-density lipoprotein receptor (LOX)-1, the same BFC has been utilized for unselective ^68^Ga-labeling of the other two nanobodies (MMR 3.49, cAbVCAM-1−5) [64,65,66], plus eight further nanobodies (2Rs15d, 4hD29, 9077, 9079, Nb109, K2, Nb1053, SNA006a) [54,67,68,69,70,71,72], with 9077 and 9079 both addressing the cell surface marker CD20 [71], Nb109 and K2 both being directed against human PD-L1 [69,70], as well as Nb1053 and SNA006a targeting CD38 and CD8, respectively [67,68]. Moreover, in GGGYK-NOTA (Figure 12), *p*-SCN-Bn-NOTA has been attached to the ε-amino group of the *C*-terminal lysine as part of the pentapeptide H-Gly-Gly-Gly-Tyr-Lys-NH_2_ in order to allow also for site-specific ^68^Ga-labeling of 2Rs15d, cAbVCAM-1−5 and K2 [18,66,69]. However, the direct comparison with their randomly-labeled counterparts revealed no significant differences with regard to targeting efficacy determined for 2Rs15d in in vitro cell binding assays, biodistribution ascertained for cAbVCAM-1−5 in ex vivo experiments, and tumor uptake investigated for K2 in in vivo studies. Besides the sortase A enzyme approach, site-specific ^68^Ga-labeling of K2 was also realized through maleimide-NOTA (Figure 12) conjugated to a cysteine engineered to the nanobody’s *C*-terminus [73]. Prior to the Michael addition reaction, however, mild reducing conditions were applied in order to particularly free the *C*-terminal thiol group from disulfides formed by dimerization or glutathione-capping, while leaving internal cysteines that are essential for the nanobody’s tertiary structure intact.

##### Acyclic

**Figure 13:**
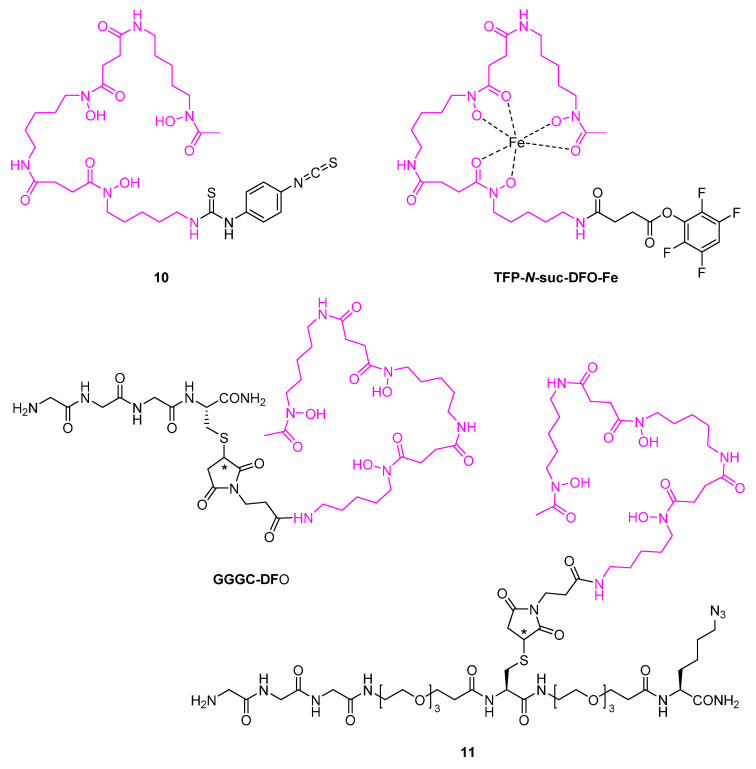
Desferrioxamine B (DFO)-based synthetic chelators used for radiometal labeling of nanobodies. DFO substructure is highlighted in pink.

The acyclic chelator desferrioxamine B (DFO) is a naturally occurring siderophore that bears hydroxamate functions for complexing radiometals [12,74]. In the form of the BFC **10** bearing a reactive isothiocyanate group (Figure 13), DFO has been randomly conjugated to primary amines of the anti-HER1 nanobody 7D12 to facilitate its radiolabeling with gallium-68 or zirconium-89 [74,75]. The same BFC has also been used for unselective ^89^Zr-labeling of the anti-gelsolin nanobody NB11 on the one hand, and of the two nanobody heterodimers 1E2-Alb8 and 6E10-Alb8 on the other hand [76,77]. In the latter two cases, nanobodies (1E2, 6E10) targeting the hepatocyte growth factor have been linked to an albumin-binding nanobody unit (Alb8) in order to extend the circulation time [76]. A similar concept has been realized in nanobody construct MSB0010853 consisting of three interconnected, mouse-human cross-reactive nanobodies, out of which one is specifically directed against albumin and two address the target HER3 at distinct epitopes [78]. While usually in post-labeling methods the chelating unit is empty when the BFC is attached to the nanobody, for pre-modification of MSB0010853, TFP-*N*-suc-DFO-Fe (Figure 13) has been applied, in which the hydroxamate groups have been temporarily blocked with the trivalent iron cation [78]. After randomly reacting its 2,3,5,6-tetrafluorophenyl ester with the construct’s primary amines, the iron was efficiently detached from DFO and subsequently labeled with zirconium-89 leading to the desired radio-probe.

Moreover, site-specific ^89^Zr-labeling has been conducted on nanobody VHH-X118 targeting the mouse cell surface marker CD8 [79,80]. Initially, the tetrapeptide-based sortase A substrate GGGC-DFO (Figure 13) was ligated to the *C*-terminus, which itself was obtained from the addition reaction between the cysteine’s thiol and the maleimide of functionalized DFO. Furthermore, with compound **11** (Figure 13), a pegylated version of the substrate was established. Therein, a carboxyl-to-amine linker containing three PEG units was introduced between the triglycine and the cysteine on the one hand, and at the cysteine’s terminal carboxamide group on the other hand, where it was further extended by a modified lysine bearing a *C*-terminal carboxamide and an ε-azide group. After selective conjugation of **11** to VHH-X118, the azide click handle allowed for the additional introduction of PEG units via SPAAC, followed by ^89^Zr-complexation. The resulting radiotracers exhibited an improved image quality compared to the non-pegylated counterpart, due to the prolonged plasma half-life along with the reduced accumulation in elimination organs. Hence, another nanobody, i.e., H11, was also site-specifically labeled with zirconium-89 by following this pegylation strategy [47].

**Figure 14:**
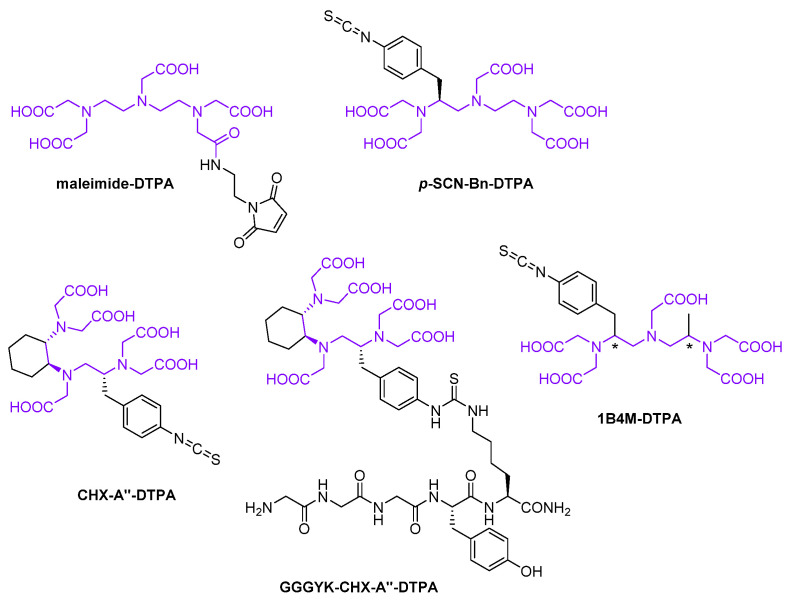
Diethylenetriaminepentaacetic acid (DTPA)-based synthetic chelators employed for labeling nanobodies with radiometals. DTPA substructure is highlighted in violet.

Diethylenetriaminepentaacetic acid (DTPA) is one of the oldest acyclic chelators, which is clinically widely established and valued for its well defined labeling techniques enabling facile and stable incorporation of indium-111 and lutetium-177 even at room temperature [81,82,83]. As part of maleimide-DTPA (Figure 14), it has been utilized for site-specific ^111^In-labeling of three nanobodies, i.e., 2Rs15d, JVZ-007 and 4hD29, with the latter two being specific to the prostate-specific membrane antigen (PSMA) and the enzyme dipeptidyl-peptidase 6, respectively [16,82,84]. For this purpose, the *C*-termini of these three nanobodies were engineered to bear a cysteine, which due to spontaneous oxidative homodimerization required mild reducing conditions to specifically free this thiol for the Michael addition reaction with the maleimide moiety on the one hand, but maintaining the intradomain disulfide bridges on the other hand. Furthermore, the BFC *p*-SCN-Bn-DTPA (Figure 14) has been randomly introduced to primary amines of JVZ-007 as well as of the amyloid-targeting nanobody VHH-pa2H, in order to allow for ^111^In-chelation [82,83]. CHX-A″-DTPA (Figure 14) represents a structural analog of *p*-SCN-Bn-DTPA, in which the non-benzyl substituted flexible ethylene backbone is fixated by a butane chain forming a six-membered ring [81]. Such cyclohexyl moiety imparts a higher degree of rigidity to the chelating unit and with that an imposed preorganization on the metal ion binding site leading to an enhanced kinetic inertness of the radiometal complex. By fusing CHX-A″-DTPA with its isothiocyanate group to the lysine’s ε-amino group of the pentapeptide H-Gly-Gly-Gly-Tyr-Lys-NH_2_ as in GGGYK-CHX-A″-DTPA (Figure 14), site-specific ^111^In-labeling of the two nanobodies 2Rs15d and cAbVCAM-1−5 has been realized [18,66]. CHX-A″-DTPA has also been directly applied to primary amines of these nanobodies for random incorporation of indium-111 [18,66,85]. However, head-to-head comparison with the selective ^111^In-tracers did not reveal a significant difference with respect to targeting efficacy of 2Rs15d and biodistribution of cAbVCAM-1−5. Based on nanobody 9077, different constructs have been developed including monomeric, homodimeric and heterodimeric structures, which were randomly decorated with CHX-A″-DTPA through their primary amines in order to allow for ^111^In- and ^177^Lu-labeling, respectively [86]. In the same way, nanobody 9079 in its monomeric form has been radiolabeled with lutetium-177 [71]. 1B4M-DTPA (Figure 14), also known as tiuxetan, is structurally even closer to *p*-SCN-Bn-DTPA than CHX-A″-DTPA, differing from it only by a single methyl group situated on the outer carbon of the non-benzyl substituted ethylene backbone [81]. This BFC has been randomly employed to primary amines of the two nanobodies 2Rs15d and R3B23 to enable ^177^Lu-chelation [59,85,87]. The latter displays a very specific nanobody, targeting the monoclonal idiotype present in the murine 5T2MM model (5T2MMid), which itself is a syngeneic immunocompetent model resembling human multiple myeloma clinically and biologically [59].

#### 2.2.2. Proteinogenic Chelator

**Figure 15:**
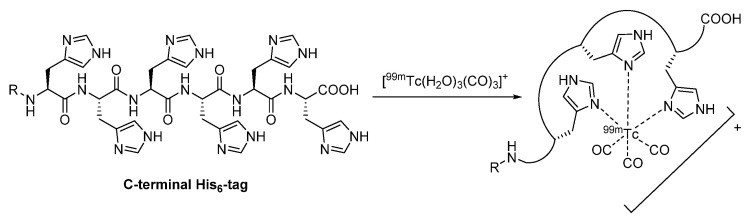
Complex formation between a *C*-terminal hexahistidine-tag and ^99m^Tc-tricarbonyl [110].

Besides the attachment of synthetic chelators and thereby chemical modification of the nanobodies’ molecular structure, a certain part of their amino acid sequence offers a very convenient and site-specific labeling technique [18]. In fact, a *C*-terminal hexahistidine (His_6_) tail is usually genetically engineered in order to facilitate the purification of the protein on a nickel affinity column [54,61], which also enables the incorporation of technetium-99m in the form of ^99m^Tc-tricarbonyl ([[^99m^Tc]Tc(H_2_O)_3_(CO)_3_]^+^) [16,88]. The tricarbonyl core is efficiently coordinated by three of the total of six histidine-derived imidazole residues, forming the basis for this elegant method (Figure 15) [61]. Since the His_6_-tag is located on the opposite side of the paratope, the antigen-binding activity remains usually unaffected [13,61]. This labeling strategy has been applied not only to a huge panel of nanobodies addressing the so far discussed targets, e.g., HER-1 [89], MMR [53,90,91], VCAM-1 [92,93], PD-L1 [94,95], CRIg [96], LOX-1 [97], gelsolin [98,99], PSMA [100], amyloid [101], but also to several nanobodies that are directed against other structures, such as carcinoembryonic antigen (CEA) [102], mesothelin [103], CD33 [88], murine bone marrow-derived dendritic cells [104], mouse C-type lectin domain family 4 member F (Clec4F) [105], and mouse lymphocyte-activation gene 3 (LAG-3) [106,107]. However, such an additional His_6_-tag implies disadvantages with respect to clinical application, comprising induction of immune responses on the one hand [108,109], and high kidney retention on the other hand [54]. Accordingly, in the context of multiple imaging modalities, ^99m^Tc-tricarbonyl-labeled nanobodies display a rather limited scope [18].

#### 2.2.3. Heteroleptic Complex

**Figure 16:**
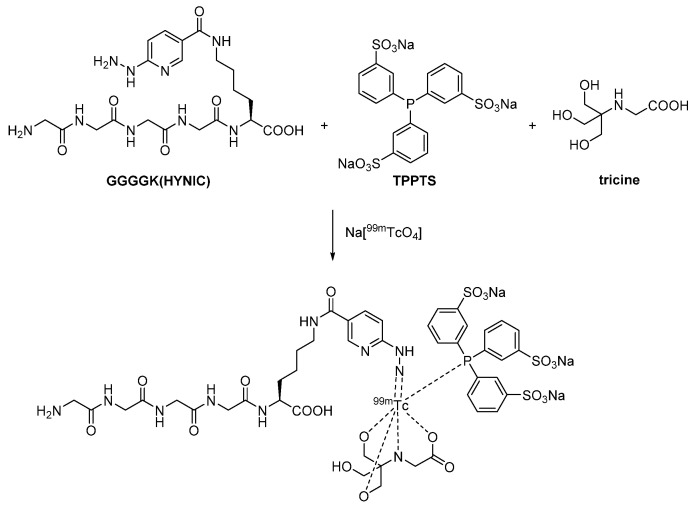
Assumed complex formation of technetium-99m coordinated by three ligands [112].

Another way to radiolabel nanobodies with technetium-99m has been described by Gao et al. [111]. Therein, prior to attachment to the murine (MY1523) and the human (NB17) PD-L1-targeted nanobody, respectively, the heteroleptic complex consisting of technetium-99m coordinated by the three ligands GGGGK(HYNIC), triphenylphosphine-3,3′,3″-trisulfonic acid trisodium salt (TPPTS) and tricine was formed (Figure 16). GGGGK(HYNIC) is based on the pentapeptide H-Gly-Gly-Gly-Gly-Lys-OH, in which the ε-amino group of the *C*-terminal lysine is acylated by 6-hydrazinonicotinic acid. While the hydrazine moiety takes part in the complex formation, the tetraglycine identifies the molecule as a substrate for sortase A, thereby enabling the label installation.

## 3. Conclusions

With the introduction of new tracers, molecular nuclear imaging has become increasingly important in recent years [113]. While success was often achieved with small molecules as radiolabeled ligands for PSMA [114], nanobody-based radio-probes are coming more and more to the fore in current research [69,115]. During the past twelve years, many different strategies for radiolabeling nanobodies have been implemented (Table 1), including both radiohalogens and radiometals, which have been introduced randomly or site-specifically to the nanobodies’ peptide chain. Among these, the most convenient method is certainly the site-specific ^99m^Tc-labeling through an engineered His_6_-tag. However, with respect to clinical diagnostics, the other techniques are much more favorable. Even though chelator-based radiolabeling of nanobodies appears to dominate currently, it still remains to be seen which of all of the approaches described herein will prevail henceforth.

Future applications of radiolabeled nanobodies may range from oncological questions, such as tumor specific receptor statuses, to the visualization of cardiovascular or neurological diseases. One of the first targets investigated was the HER2 receptor status in breast cancer patients, which is a crucial point for the treatment of these patients when suffering from advanced disease [27]. In the context of receptor status in oncology, heterogeneity is also an important topic and an essential question for molecular imaging, as this cannot be assessed by biopsy of single lesions, which is nowadays often used for treatment planning [116]. In addition, in many other tumor entities, heterogeneity seems to be a key factor in connection with treatment planning, as e.g., in melanoma [117], or as a potential surrogate marker in liver tumors or liver metastases [118]. Accordingly, it is also a major point to examine tumor heterogeneity in other pathological markers such as PD-L1, which also represents a pivotal target for nanobody-based imaging [95]. This, of course, needs to be discussed in association with quantitative molecular imaging and its limitations such as spatial resolution and the need for standardization of scanners and imaging protocols [119]. Moreover, immunological processes can be investigated in much more detail with radiolabeled nanobodies than with conventional radiotracers, which are certainly able to provide some basic information [120], but are limited in specificity. This is also of great interest in terms of cardiovascular diseases [121].

All in all, nanobodies seem to constitute a powerful and safe tool for the development of new radiopharmaceuticals for various applications. For imaging purposes, there is a high variety of intriguing labeling strategies, as outlined in this review.

## Figures and Tables

**Table 1:** Pros and Cons of the herein discussed radiolabeling techniques applied to nanobodies.

Radiolabeling Strategies		Positive Aspects	Negative Aspects
**Radiohalogens**	Iodogen	sufficiently mild method to directly radioiodinate sensitive proteins at low temperatures	solely unspecific labeling possible limited to radioiodines
	Prosthetic groups	labeling with different radiohalogens feasible huge variety in their design realizable	their preparation and purification are usually sophisticated resulting in a long labeling procedure and low chemical yields
	Chelation	simplified labeling process leading to good radiochemical yields as well as high molar activities	only applicable to fluorine-18 radioactivity is not introduced in the final step
**Radiometals**	Synthetic chelators	radiolabel is inserted at the very end different radiometals are introducible to the same chelator	consistently metal-free conditions essential can affect the physicochemical properties of the nanobody
	Proteinogenic chelator	chelator is often engineered for purification reasons enables site-specific labeling with technetium-99m	induction of immune responses ^99m^Tc-tricarbonyl ^#^ required, which has to be prepared
	Heteroleptic complex	no conversion of technetium-99m necessary	radiocomplex needs to be formed prior to attachment

^#^ [[^99m^Tc]Tc(H_2_O)_3_(CO)_3_]^+.^

## Data Availability

Not applicable.
